# Bioactive Properties of Bread Formulated with Plant-based Functional Ingredients Before Consumption and Possible Links with Health Outcomes After Consumption- A Review

**DOI:** 10.1007/s11130-022-00993-0

**Published:** 2022-07-20

**Authors:** Isaac Amoah, Carolyn Cairncross, Emmanuel Ofori Osei, Jacqueline Afua Yeboah, Jesse Charles Cobbinah, Elaine Rush

**Affiliations:** 1 Faculty of Health and Environmental Sciences, Auckland University of Technology, Auckland, 1010 New Zealand; 2 Riddet Institute, Massey University, Private Bag 11222, Palmerston North, 4442 New Zealand; 3 Department of Biochemistry and Biotechnology, Kwame Nkrumah University of Science and Technology, Kumasi, Ghana

**Keywords:** Functional bread, Health effect, Health claims, Physiological effect, Phytochemicals, Bioactivity

## Abstract

Bread is a commonly consumed staple and could be a viable medium to deliver plant-based ingredients that demonstrate health effects. This review brings together published evidence on the bioactive properties of bread formulated with plant-based ingredients. Health effects associated with the consumption of bread formulated with plant-based functional ingredients was also reviewed. Bioactive properties demonstrated by the functional ingredients fruits and vegetables, legumes, nuts and tea incorporated into bread include increased phenolic and polyphenolic content, increased antioxidant activity, and extension of bread shelf-life by impairment of lipid and protein oxidation. Acute health effects reported included appetite suppression, reduced diastolic blood pressure, improvements in glycaemia, insulinaemia and satiety effect. These metabolic effects are mainly short lived and not enough for a health claim. Longer term studies or comparison of those who consume and those who do not are needed. The incorporation of plant-based functional ingredients in bread could enhance the health-promoting effects of bread.

## Introduction

The formulation of bread with plant-based functional food ingredients can enhance its bioactive properties. For example, ingredients that increase polyphenol and carotenoid concentrations increase the antioxidant properties of the bread [1]. For wholegrain breads such as rye that have oil added, shelf-life and texture of the bread are improved but potential for lipid oxidation is increased [2, 3]. The addition of bioactive compounds from plant-based functional ingredients to these breads may inhibit lipid and protein oxidation and also slow the growth of molds and slow bread spoilage [4]. What is not known is whether these increased bioactive properties in the bread matrix will result in improvements in markers of health and reduction of risk for disease after the bread is consumed.

Globally, refined bread is a commonly consumed staple food [5]. The basic ingredients for bread formulation are white wheat flour, yeast and water. Wheat flour is an essential ingredient for most breads due to the provision during bread dough development of a viscoelastic network by the gluten proteins, glutenin and gliadenin [6]. Refined flours store well compared with wholemeal flours because the nutrient dense germ and bran are removed and the kernel has minimal dietary fibre and few nutrients. However, traditional white bread, is a high glycaemic index (GI) food contributing substantially to the glycaemic load (GL) of diets that contain a large amount of bread. When consumed, white bread is associated with a higher rate of postprandial glucose release and lower satiety sensations than breads formulated with whole meal [7]. These are factors that are strongly associated with the development of type 2 diabetes mellitus [8]. Consequent to this, white bread could be targeted as an appropriate medium for reformulation and the delivery of plant-based ingredients both to increase nutrient density, slow post-prandial glucose release and offer possible functional benefits of phytochemicals that may regulate human metabolic functions and have beneficial effects to health [1, 9].

Many phytochemicals such as carotenoids, phenolic acids, tannins, stilbenes, lignin, and coumarins demonstrate bioactivity including inhibition of the activity of *α*-amylase enzyme slowing the digestion of carbohydrate [10]. However, several of the potential health-effects associated with bioactive compounds present in plants have been tested using *in vitro* models without validation in human trials [11–13].

We recently reviewed the prospect of incorporating flours from plant-based food by-products such as bran and seeds into bread and reported the potential for enhancement of the nutritional, bioactive and health effects when consumed [14]. This review addresses the potential for use of whole-plant-based ingredients and extracts in bread products. Plants examined include fruit and vegetables, seeds herbs and leaves and tubers rhizomes. Potential beneficial health effects associated with the consumption of functional bread formulated with plant-based functional food ingredients was also reviewed.

## Search Strategy

The authors rigorously searched the databases Web of Science, PubMed and the Cochrane library. The search terms employed were: 1. (Bread OR Loaf) AND (Novel ingredient*) 2. (Bread OR Loaf) AND (Phytochemical*) 3. (Bread OR Loaf) AND (Bioactive* compound*) 4. (Health*) AND (Bread OR Loaf) 5. (Health*) AND (Bread OR Loaf) AND (Develop*) 6. (Health*) AND (Bread OR Loaf) AND (Health effect*) 7. (Health*) AND (Bread OR Loaf) AND (Functional) 8. (Health*) AND (Bread OR Loaf) AND (Functional) AND (Health claim*) 9. (Health*) AND (Bread OR Loaf) AND (Fortify* or enrich*) 10. (Health*) AND (Bread OR Loaf) AND (Fortify* or enrich*) AND (Health claim*) 11. (Bread OR Loaf) AND (Physiological effect* OR Physiological action* OR Physiological function*) 12. (Bread OR Loaf) AND (Medicinal herb* OR Herbal extract*).

A critical review of the articles extracted from the databases used above showed that no previous article has reviewed both the bioactive properties of plant-based ingredients utilised in bread formulation and established the potential health effects associated with the consumption of these ingredients. This highlights the novelty of this present review work.

## Bioactive Properties of Breads Enriched with Plant Ingredients and Extracts

### Fruits and Vegetable Enrichment of Bread

Fruits and vegetables are a rich source of essential bioactive compounds including carotenoids and polyphenols which have beneficial health properties [15]. Consequently, public health promotion messages advocate for increased fruit and vegetables intake in many countries including New Zealand with the message 5 + Aday [16]. The global need to shift to more sustainable diets with a lower carbon footprint including fruits and vegetables is another reason for the promotion of increased fruit and vegetables consumption. Factors that limit fruits and vegetable consumption include a short shelf-life, lack of availability and access during off-seasons and cost [17]. Fresh fruits and vegetables do not store well due to their high moisture content and water activity [18]. Therefore, to promote the consumption of fruit and vegetables during off-season and reduce wastage requires processing the dried produce into flour for later use in food product development. Commonly processing methods that could be employed for the processing of fruit and vegetables into flour that has a higher storage property include the use of freeze-drying, drum-drying and oven-drying [14]. Examples of fruit and vegetable flours used in bread formulation and the bioactive properties demonstrated by these ingredients in the bread matrix are reported below.

Bread supplementation with flour from the fruit of doum (*Hyphaene thebaica *L.) [11], *Garcinia mangostana* pericarp [13], baobab fruit [19], saskatoon berry [12], acorn [20], albedo fruit [21], sea buckthorn berry [22] and staghorn sumac [23] resulted in enhanced concentrations of polyphenolic [19], total phenolics [11–13, 20, 21, 23], anthocyanin [23] and flavonoid content of the supplemented bread [11]. Additionally, an increased antioxidant activity was reported for many of the breads [11–13, 21, 22]. In most of the studies reported above, the increased antioxidant properties demonstrated by the bread were positively associated with the dose of plant-based functional ingredients in the bread. The enrichment of bread with flour produced from vegetables (carrot, tomato, beetroot and broccoli flour) resulted in the favourable effect of impaired lipid and protein oxidation, respectively [2, 3]. Additionally, bread enrichment with these vegetables resulted in increased antioxidant content. The resultant effect was longer shelf life of the bread when compared to their non-enriched counterparts especially when powders or flours made from grinding dried plants such as beetroot and broccoli were utilised for the formulation [2, 3]. Similar to the reports cited above, bread enriched with functional ingredients including cladodes powder [24], broccoli sprouts [25], red bell peppers [26], onion skin [27] and *Amaranthus viridis*, *Solanum macrocarpon*, *Telfairia occidentalis* [28] recorded increased polyphenolic and total phenolic content, antioxidant potential [24–28], content of flavonoids [26] and *in vitro,* improved protein digestibility [27] of each of the enriched breads. Ranawana et al*.* [3] interestingly observed that wheat flour bread containing corn oil as an ingredient and enriched with freeze-dried carrot, tomato, beetroot or broccoli flours improved nutrient density, antioxidant properties and particularly for beetroot and broccoli increased storage life compared to their oil-free counterpart [2]. This could be due to the fat soluble bioactive compounds including carotenoids dissolving in the oil with the result of increased concentration of carotenoids therefore increased antioxidant activity [29].

### The Utilization of Oilseed/Tree Plant Seed/Extract, Legume/Bean as Functional Ingredients in Bread-making

Seeds, including oilseeds, and legumes are rich sources of polyunsaturated fatty acids and other essential bioactive vitamins such as *α*-tocopherol which is also an antioxidant [30]. In bread-making, the addition of fats and oils to the dough imparts acceptable textural attributes to the bread and improves its sensory profile [31]. Thus, the addition of oil seed functional ingredients in bread could have a dual role of not only enhancing the organoleptic properties of the bread but will additionally deliver essential bioactives and nutrients in the bread matrix [30]. The addition of crushed seeds to dough increases the oil and bioactive content of the dough. For example, whole soybean seed has 17.5% fat [32] and flaxseed contains 41% fat [33]. Despite the addition of oil to the bread dough matrix improving textural properties of bread, bread with a higher fat content may be susceptible to spoilage due to fat oxidation. However, the addition of crushed oil seeds but not finely milled could minimize the degree of deterioration as the oil remain bound in the cell walls of the seeds. The seeds of flax [34], fenugreek [35], fennel [36], *Perilla frutescens* [37] and their extracts have been used in bread formulation and their bioactive effects in the bread matrix reported. Enrichment of bread with powders from the seeds enhanced the concentration of phenolics [34–37], flavonoid [35], *α*-linolenic acid [37] and improved the antioxidant properties of the bread [35–37]. In most cases, the concentration of the bioactive compounds reported for the bread was related to the dose of seed flour in balance with the desirable physicochemical characteristics. For example, bread enriched with fennel seed powder reached an optimized total phenolic concentration and antioxidant effect with the addition of 7% fennel seed powder [36]. In the case of its 2,2-diphenyl-1-picrylhydrazyl (DPPH) scavenging ability, optimization was attained with a 5% fennel seed powder enrichment [36]. The sustainable development goals 2 highlights the need to achieve Zero hunger at the population level [38]. Again, the need to promote the utilisation of alternative protein sources such as nitrogen-fixing legumes is a key way to achieve the SDG goal. For example the enrichment of wheat bread with lupin resulted in increased concentration of protein, carotenoids, polyphenolic, and antioxidants concentration and protein quality was improved [39]. A similar increase in phenolic concentration and improved antioxidant activity was reported for bread enriched with green coffee bean [40]. The predominant phenolics present in the green coffee bean-bread were caffeic acid, syringic acid and vanillic acid [40].

### Herbs/Weed and Leaf Extract Supplementation in Functional Bread

Edible plants such as herbs and spices are an active area for research in relation to their rich phytochemical composition and their possible advantageous contribution to dietary variety and diversity [41]. For example, green tea is rich in the polyphenols catechin, epicatechin, epicatechin gallate and epigallocatechin [42], black tea contains the flavanols theaflavin, theaflavin-3-gallate and thearubigin [42], white mulberry leaf is rich in flavonoids, phenolics and ascorbic acid and beta carotenes [43] and *Potentilla anserina* contains triterpenes [44]. Bioactive properties reported for the enriched breads included enhanced antioxidant properties [45–48] as a result of the increased total phenolic content [46, 49] and polyphenolic concentrations [47]. The formation of lipid hydroperoxides in bread enriched with herbs and leaves is slowed [48]. Examples of slowed spoilage include bread enriched with black tea [45], leaf extract of white mulberry [47], green tea powder [48], *Potentilla anserina* [49], and theanine/polyphenol fractions from black tea dust (decaffeinated) [46]. There was an observation of a dose-dependent total phenolic and antioxidant increase observed for bread enriched with polyphenolic compounds produced from decaffeinated black tea dust [46] and yerba mate leaves [50]. In a study that involved bread enrichment with powder produced from the root of Shatavari (*Asparagus racemosus*), the presence of secondary plant metabolites including saponin, alkaloid, steroid and terpenoid [51] which may potentially demonstrate some physiological effects that was observed.

### Tubers and Rhizomes Incorporation in Bread as Functional Ingredients

The incorporation of flour and flour-based products from the tubers of purple potato [52], yam (*Dioscorea purpurea*) [53], into functional bread resulted in enhanced antioxidant properties [53] of the bread. More specifically, the concentration of free phenolic acids (gallic, chlorogenic acid, protocatechuic, caffeic acid, vanillic, ferulic acid, *p*-coumaric acid and syringic acids) and antioxidants increased in purple potato powder-enriched bread compared to its yellow potato counterparts [52]. That notwithstanding, bread enrichment with increasing quantities of yellow potato flour (3, 6 and 9%) increased *β*-carotene concentration and bread quality [54]. The antioxidant properties of bread enriched with powder from turmeric, a rhizomatous plant, was enhanced when compared to its non-turmeric enriched counterpart [55].

While the research reviewed above demonstrates that fortification of breads with diverse plant products can enhance physicochemical properties including antioxidation, increase concentrations of diverse phytochemicals, extend shelf-life and prevent wastage of edible plants the assumption that the presence of the phytochemicals in the bread at the mouth translates into health benefit must be tested. The next step is to review effects of these fortified breads on human metabolism associated with the consumption of bread.

## Metabolic Effects Associated with Consumption of Functional Bread

The clinical studies that examined the metabolic effects associated with the consumption of functional bread were acute with outcomes measured hours or a few days after consumption (Table 1). Treatments were randomised and cross-over with relatively short wash-out periods.

**Table 1:** Acute effects on health outcomes of addition of functional food ingredients to bread

Functional food ingredient	Type of study	Minutes of digestion	Number of subjects	Dose of bread served & bioactive where provided	Health effects	Country of study	Reference
Fruit and vegetables							
Baobab fruit extract	Randomised crossover study	180 min	13 healthy normal or slightly overweight volunteers	50 g of available carbohydrate	Lower postprandial insulin release	United Kingdom	[56]
Beetroot	Acute, randomised, open-label, controlled crossover study	420 min	23 healthy men	200 g bread containing 100 g beetroot (1.1 mmol nitrate) or 200 g control white bread (CB; 0 g beetroot, 0.01 mmol nitrate)	Lower diastolic blood pressure	United Kingdom	[57]
Legumes, seeds and nuts							
White kidney bean extract (*Phaseolus vulgaris*)	Randomised crossover study	120 min	13 healthy adults	Dosages of 1500 mg, 2000 mg, and 3000 mg kidney bean extract	Lower postprandial glucose release	United States of America	[58]
Chickpea	Randomised, crossover study	90 min	13 healthy female subjects	50 g supplying 25 g available carbohydrate	Lower postprandial glucose release	Kuwait	[59]
Chickpea	Randomised, single-blind, crossover study	120 min	11 healthy subjects	50 g available carbohydrate	Lower postprandial glucose release	Australia	[60]
Lupin	Randomised controlled crossover study	180 min	16 and 17 respectively for study 1 and 2	40 g total carbohydrate	Increased satiety, energy intake lowered	Australia	[61]
Lupin	Randomised crossover study	120 min	20 healthy adults	36.9 and 37.3 g available carbohydrate for Burgen and Lupin breads respectively	Lower postprandial glucose release and increased satiety	Australia	[62]
Australian sweet lupin (*Lupinus angustifolius*) kernel fibre	Randomised crossover study	120 min	21 healthy adults	50 g available carbohydrate breakfasts/ 90 g lupin kernel flour	Lower postprandial insulin release	Australia	[63]
Salba	Randomised controlled crossover study	120 min	13 healthy adults	7, 15 or 24 g of whole or ground Salba baked into white bread	Lower postprandial glucose release	Canada	[64]
Salba	Acute randomised, double-blind, crossover study	120 min	11 healthy subjects	50 g available carbohydrate with addition of 0, 7, 15 or 24 g of Salba	Lower postprandial glucose release and improved appetite response	Canada	[65]
Sliced hazelnut and semi-defatted hazel nut flour	Randomised controlled, crossover study	6 days-120 min	32 healthy adults	Breads contained either 30 g of finely sliced hazelnuts, 30 g semi-defatted hazelnut flour, or 15 g of each (amounts per 120 g bread)	Lower postprandial glucose release	New Zealand	[66]
Gum							
Guar gum	Randomised crossover study	240 min	40 healthy adults	50 g available starch	Cognitive function and glucose response improved	Sweden	[67]
Medium weight guar gum	Randomised crossover study	180 min	12 healthy volunteers	37 g of available starch	Glucose, insulin and subjective appetite ratings improved	Sweden	[68]
Guar gum	Randomised crossover study	240 min	19 healthy adults	50 g of available starch	Appetite improved	Sweden	[69]
Guar gum	Randomised crossover study	120 min	17 healthy subjects	50 g ‘available’ carbohydrate	No significant differences in the post-prandial blood glucose responses reported after the intake of the control and guar breads. Post-prandial rise in plasma insulin was blunted by the intake of all the guar breads	England	[70]

### Fruit and Vegetable-enriched Bread and Health-effects Associated with its Consumption

The section preceding this reviewed the bioactive properties of bread enriched with fruit and vegetable ingredients. In a recent systematic review and meta-analysis of prospective studies [71], the authors reported that increased fruit and vegetable consumption was strongly associated with a lower incidence of morbidity and mortality from chronic diseases including cancer and cardiovascular disease. The health effects demonstrated by the fruits and vegetables are attributed to their nutrient density and content of diverse bioactive compounds including polyphenols and carotenoids [72, 73]. Baobab fruit due to its rich polyphenol composition could inhibit the activity of carbohydrate digestion enzymes thus potentially improving glycaemic response status [10]. However, bread enriched with flour from baobab fruit induced a substantially reduced postprandial insulin release but not glucose when consumed [56]. This could be attributed to the rich fibre (pectin) composition of baobab fruits. We recently reported a similar finding for bread enriched with drum-dried pumpkin and sweetcorn flour [74]. The consumption of the bread enriched with the vegetables pumpkin and sweetcorn showed no changes in glucose concentration but rather resulted in a lower insulin release when compared to white and wheatmeal breads [74]. Several systematic reviews of randomised controlled trials have reported an association between beetroot juice consumption and improved blood pressure parameters [75]. This has been attributed to the rich nitrate content of beetroot which is converted to nitric oxide when consumed, a potent vasodilator [76]. Hobbs et al. [57] enriched bread with beetroot flour and investigated its effect on blood pressure parameters. The authors reported that, the beetroot bread intake resulted in decreased diastolic blood pressure (iAUC of -13.0 mmHg × h) but not for systolic blood pressure [57] (Table 1).

Despite several studies on bread enrichment with fruits and vegetables and their associated bioactive properties reported in the preceding sections, there remains few articles that have clinically validated their health effects in humans (Table 1). This highlights the apparent disconnect between mainstream food science and technology and human nutrition. Food science and technologists have traditionally focused on the development of bread enriched with fruits and vegetable flour and conducting acceptability studies to evaluate its liking amongst consumers at the neglect of validating the health-promoting benefits through clinical trials. There is the need for a new paradigm in research and development regarding bread production where human nutritionists with expertise in health effects validation in bread products could work with food scientists to step up the developments of bread enriched with plant-based functional ingredients that have appealing organoleptic properties and demonstrates health-promoting properties.

### Legumes, Seeds and Nuts in Bread and Health-effects Associated with its Consumption

Legumes, seeds and nuts consumption is encouraged as they are a rich source of essential bioactive compounds and fibre [77] and is associated with improved glycaemic and lipid profile status, (Table 1) which may be attributed to factors including increased phenolic composition which impairs amylase enzyme activity [10]. Bread enriched with flours from chick pea [59, 60], white kidney bean extract [58], lupin [61–63] and salba [65] when consumed demonstrated a lower postprandial glucose and insulin release compared to its non-enriched counterparts [59, 60, 62, 63, 65], and in two trials improved subjective appetite sensation were measured [61, 65]. In addition to the bioactive composition of legumes, nuts and seeds, the structural properties of these ingredients may potentially impact on their health-promoting effects. To investigate this, whole and ground flour from the seeds of salba (*Salvia hispanica *L.) was used to enrich bread and its glycaemic properties investigated through consumption of the bread [64]. The authors reported that, no difference in postprandial glucose release was recorded when either the whole or ground salba seed-enriched bread was consumed [64]. This observation was also reported for hazel nut-enriched bread [66]. The authors incorporated finely sliced hazelnut and semi-defatted hazelnut flours into bread and determined its glycaemic properties. They reported that, postprandial glucose release was significantly attenuated for all nut-enriched breads but subjective satiety sensations were not different [66]. In terms of acceptability, bread enriched with chickpea flour [59, 60] was not significantly different from their control white bread [60] and whole wheat bread counterpart [59]. The incorporation of bread with Australian sweet lupin resulted in no significant acceptability difference from its control counterparts [63]. Devi et al. [66] enriched bread with sliced hazelnut and semi-defatted hazel nut flour. The acceptability of the sliced hazelnut enriched bread was significantly higher than its control counterpart. However, the acceptability of the semi-defatted hazel nut flour-enriched bread was not different from the control counterpart [66].

For some individuals, consumption of legumes, pulses and nuts may cause gastrointestinal discomfort including bloating. Consequently, the gastrointestinal and physical discomfort associated with the consumption of bread enriched with white kidney beans, chickpea flour and hazelnut were subjectively evaluated using a 10-point Likert scale and visual analogue scale (VAS). The assessment questions included “flatulence”, “belching”, “stomach bloating”, “stomach cramping”, “flatulence”, “diarrhoea”, and “stomach ache”[58, 66], (“How well do you feel?” with options such as “not well at all” or “very well” given at the opposite ends of the line [59]. The remainder of the studies did not investigate the toxicological aspects of the functional breads developed nor did any study report consumer evaluation of sensory attributes and acceptability. This could be partly attributed to the fact that the ingredients used in the bread formulation are well known wholesome plant foods consumed by people as part of their normal diets. These include beetroot and baobab.

The seeds of the leguminous crop *Cyamopsis tetragonolobus* are the raw material for guar gum production [78]. Guar gum is a food fibre that can act as a hydrocolloid when used in bread enrichment and can subsequently improve the softness of the bread crumb [79]. Guar gum demonstrates health effects including improved glycaemic response and decreased absorption of lipids and improved laxation [79]. Bread with relatively soft crumb properties is generally accepted by consumers and could be used as a medium of delivery for older adults with chewing and swallowing challenges. In our recently published study on vegetable (pumpkin and sweetcorn) enrichment with bread, we recruited older adults to conduct sensory and ease of swallowing evaluation of the bread [80]. The participants subjectively indicated that, bread enriched with the vegetables was easier to chew and swallow compared to commercially produced wheatmeal and white breads [80]. This was attributed to the presence of pectin soluble fibres from pumpkin in the vegetable enriched bread which maintains hydration properties of the bread matrix. Guar gum in a similar manner as pumpkin flour as a fibre has good hydration properties. The inner part of the guar seed, *Cyamopsis tetragonolobus*, consists of the complex polysaccharide, galactomannan [78]. Galactomannan is made of the monomers galactose and D-mannose and thus has hydroxyl groups that by hydrogen bonding are able to interact with water molecules [78]. The consumption of guar gum-enriched bread resulted in postprandial glucose moderation with improved cognitive performance [67] (Table 1). Guar gum of medium weight was used to enrich whole grain corn flour that has increased amylose composition and used for bread formulation. The consumption of the bread resulted in significant reductions in postprandial glucose, insulin and improved subjective appetite sensations attributed to the enhanced resistant starch concentration from the guar gum and whole grain corn flour [68]. A similar observation was recorded for guar gum, whole grain rye with or without high amylose maize starch in white bread, though no impact on the release of PYY was reported [69]. No significant differences in the post-prandial blood glucose responses were observed between the guar and white bread, and all guar breads significantly induced decrease in the post prandial rise in plasma insulin, which were found not influenced by large variations in particle size of guar gum or molecular weight [70]. In the case of guar gum enriched bread, the acceptability of the bread was not different from the control counterpart except the bread enriched with guar gum with molecular weight of 150 (M150) [70].

## Strengths and Limitations

The strength of this review is that this is the first time a review has combined evidence for the bioactive properties of bread enriched with plant-based ingredients with evidence for the ability of plant-based ingredients to demonstrate health-promoting properties in short term human trials. This review found that extensive characterisation of plants in breads for human consumption has taken place at the bench with some *in vitro* modelling of digestion. However, the trials of health effects were limited to short term trials looking only at acute changes in metabolism and physiology. The dosage of the bioactive in the functional ingredient was not reported rigorously especially in the studies of glycaemic response which rely on a standard ‘available’ carbohydrate dose. In addition, the bread formulation and the proportions or combination of ingredients in the dose of bread are not standardised. For example, the quantity and quality of fibre could interact with the action of the bioactive molecules. The profiling of bioactive compounds from oil seed/tree plant seed/extract, legume/bean was limited in that most of the authors focused on only the secondary plant metabolites and did not profile all the fatty acids or measure the bioactivity of specific antioxidants in the oils from these plant sources. Furthermore, the dose of bioactive compounds should be more rigorously controlled and reported. For example, the dose could be relative to body mass and comparisons made between the response of men and women. This points out some gaps and areas for future research.

## Conclusion

This review brings together published evidence for plant-based ingredients and their bioactive properties that may contribute to the functionality of bread when consumed through increased phenolic and polyphenolic content, increased antioxidant activity, and extension of bread shelf-life by impairment of lipid and protein oxidation. Acute effects reported included appetite suppression, reduced diastolic blood pressure and improvements in glycaemia, insulinaemia, and antioxidant status of blood. The incorporation of plant-based functional ingredients in bread could enhance the health-promoting effects of bread and reduce some food waste. Future work in this area should also assess the sustainability and availability of plant-based ingredients for functional breads.

## Data Availability

There is no available data or extra materials for this review.
